# Fabrication of MoS_2_/C_60_ Nanolayer Field-Effect Transistor for Ultrasensitive Detection of miRNA-155

**DOI:** 10.3390/mi14030660

**Published:** 2023-03-15

**Authors:** Youqiang Xing, Yun Wang, Lei Liu, Ze Wu

**Affiliations:** School of Mechanical Engineering, Southeast University, Nanjing 211189, China

**Keywords:** ultrasensitive detection, FET, MoS_2_/C_60_ composite nanolayer

## Abstract

As a major public health issue, early cancer detection is of great significance. A field-effect transistor (FET) based on an MoS_2_/C_60_ composite nanolayer as the channel material enhances device performance by adding a light source, allowing the ultrasensitive detection of cancer-related miRNA. In this work, atomic layer deposition (ALD) was used to deposit MoS_2_ layer by layer, and C_60_ was deposited by an evaporation coater to obtain a composite nanolayer with good surface morphology as the channel material of the FET. Based on the good absorption of C_60_ by blue-violet light, a 405 nm laser was selected to irradiate the channel material, improving the function of FET biosensors. A linear detection window from 10 pM to 1 fM with an ultralow detection limit of 5.16 aM for miRNA-155 was achieved.

## 1. Introduction

Since the preparation of single-layer graphene in 2004, the field of two-dimensional materials has continued to develop, inspiring the field of biosensor detection. Based on the excellent physicochemical, electrical, photoelectricity, and other properties of two-dimensional materials, humans have developed new biological detection sensors such as fluorescence signal detection, electrochemical detection, and photoelectric chemical detection [1,2,3,4]. Biosensors based on field-effect transistors (FETs) have attracted much attention as they offer rapid, inexpensive, and label-free detection [5]. As a major public health issue, early screening, diagnosis, and treatment of cancer are of great significance in improving the five-year survival rate and quality of life of cancer patients [6]. Compared with traditional detection methods, the ultrasensitive detection of tumor markers in blood has great potential and application prospects in the early diagnosis and screening of cancer because of its simple and non-invasive advantages. MiRNA-155 has been proven to be directly involved in the development of human cancer. In the development of lung and breast cancer, it plays a very important role in regulating the occurrence and migration of tumors [7,8].

The field effect transistor (FET) with two-dimensional material as a channel can detect analytes quickly and sensitively through effective charge carrier interface transfer, so it has more applications in sensing detection [9]. Sarkar et al. applied FET to the detection of PH value and streptavidin [5]; An et al. used FET to detect trivalent arsenic ions [10]; Qiu and Liu et al. used FET to carry out nanopore sequencing and concentration detection of DNA [11,12]. Since biological detection is of great significance in medical diagnosis, national security, forensic medicine, and environmental monitoring, it is of great significance to design high-performance FETs for the ultrasensitive detection of biomolecules [13].

Mueller et al. prepared graphene field effect tubes for photoelectric detection [14]; Kis et al. reported the first single-layer MoS_2_ FET in 2011, which has an ultra-high switching ratio of 10^8^, a mobility of more than 200 cm^2^/V·s, and a sub-threshold slope as low as 74 mV/dec [15]; Huang et al. reported that the black phosphorus FET has a switching ratio of 10^5^ and a mobility of 1000 cm^2^/V·s [16]. Graphene has high mobility, but its zero band gap structure leads to a low switching ratio, which limits its further application in nanoelectronics and optoelectronics. Transition metal chalcogenides have a higher switching ratio and lower mobility. The mobility and switching ratio of black phosphorus are generally between graphene and transition metal chalcogenides. The channel materials of FET, the above two-dimensional semiconductors, have limits on switch ratio and mobility, which limit the sensitivity and detection limit of biological detection. Furchi et al. reported a van der Waals heterojunction FET [17]. The heterojunction barrier is used to reduce the off-state current, and the photoelectron generated by the heterojunction photoelectric response increases the carrier mobility, which greatly improves the device performance and is conducive to improving the biological detection performance.

The Van der Waals heterostructure is composed of two-dimensional materials with different energy bands stacked in a specific order, which will form electron transfer and potential barriers after contact. This barrier can quickly separate photogenerated charges, which can effectively improve the photoelectric performance of the device and can provide more carriers when applied to FETs. At the same time, the heterojunction barrier can be controlled by the applied voltage to achieve the purpose of controlling the device’s properties [17]. Wang et al. used MoS_2_ quantum dots to detect miRNA-155 with a detection limit of 7.19 fM [18]; Majd et al. used MoS_2_-FET to detect miRNA-155 with a detection limit of 0.03 fM [19]. Using MoS_2_ heterojunction as channel material to make the FET can provide a better device basis for detecting miRNA, thus obtaining a better detection limit.

In this work, based on MoS_2_ with excellent electrical and optical properties and C_60_ with good photo charge transfer properties, a MOS FET with MoS_2_/C_60_ heterojunction nanolayer as the channel material was designed and applied to the ultrasensitive detection of cancer-related miRNA-155. The detection range is 10^−11^–10^−15^ M, and the detection limit is 5.16 aM.

## 2. Experimental Details

The growth of MoS_2_ was based on the previous work of our laboratory [20]. The growth temperature zone was 400 °C. The source bottle was heated to 110 °C. After 1 h of heat preservation, the ALD cycle started. MoCl_5_, N_2_, H_2_S, and N_2_ were alternately introduced into the chamber. The pulse times were 1 s, 60 s, 1 s, and 60 s, respectively. N_2_ flow was 50 sccm. An amount of 5 mg of C_60_ powder was put on the tungsten boat of the thermal evaporation instrument, the vacuum chamber was 9.0 × 10^−4^ Pa, applying current to the tungsten boat. The evaporation current was 50 A, and the evaporation rate was 0.6 Å/s.

A MicroNano D-5A atomic force microscope (AFM) was used to obtain the surface morphology image of the nanolayer. The crystal structure of the composite films was observed by an X-ray diffractometer (XRD, Rigaku K/max-γA) with a scanning rate of 0.02°/s. Raman measurements were performed on a NANOBASE high-resolution confocal Raman microscope. Raman test conditions: 40× Objective lens (NA: 0.75), 532 nm excitation laser, 10% laser power, 8 s exposure time.

The mask was spin-coated with positive photoresist (RZJ-304), exposed for 1 s, soaked in positive photoresist developer (RZX-3038) for 60 s, and washed and dried. The evaporation electrode was selected to place 0.1 g Au particles on the tungsten boat of the evaporation instrument, the vacuum was up to 9.0 × 10^−4^ Pa, the current applied was 70 A, and the evaporation rate was 2 Å/s. A positive photoresist stripping solution (RBL-3368) was selected to soak for 8 h, and 90 Hz ultrasonic cleaning for 15 s was used to peel off the remaining photoresist. We prepared the FET device, then the transfer characteristic curve of the FET was measured with a four-point probe table.

Au NPs were also evaporated with the thermal evaporation instrument. The vacuum was 9.0 × 10^−4^ Pa, apply current was 70 A, and the evaporation rate was 0.6 Å/s. TE buffer solution containing 1 uM Probe RNA was dropped onto the FET channel with evaporated Au NPs. It was incubated at room temperature for 4 h. After the PBS was washed and dried, PBS buffer solution containing miRNA-155 of different concentrations was dropped onto the channel and incubated at 37 °C for 3 h for a four-point probe test. The additional 405 nm blue-violet light was 60 cm above the channel, and the power was 2 mW.

The sources of materials used in the experiment are indicated in Appendix A. The relevant nucleic acid sequences of target miRNA-155, probe miRNA-155 and single-base mismatch miRNA-155 are indicated in Appendix A.

## 3. Results and Discussions

### 3.1. FET Based on MoS_2_/C_60_ Heterojunction Nanolayer

**Figure 1:**
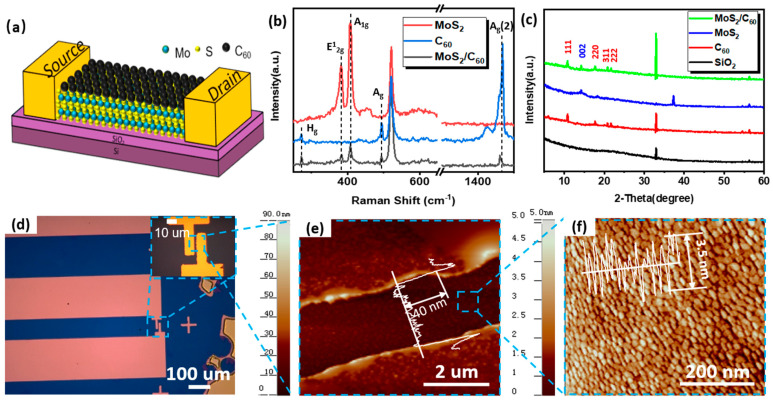
(**a**) Structure diagram of MoS_2_/C_60_ heterojunction nanolayer FET; (**b**) Raman characterization spectrum of 90c-MoS_2_, 120s-C_60_, 90c-MoS_2_-120s-C_60_; (**c**) XRD characterization of SiO_2_ substrate, 90c-MoS_2_, 120s-C_60_, 90c-MoS_2_-120s-C_60_; (**d**) Optical microscope diagram of FET device; (**e**) AFM diagram of FET device channel area; (**f**) AFM diagram of 90c-MoS_2_-120s-C_60_-FET device channel.

The structure of MoS_2_/C_60_ heterojunction nanolayer FET is shown in Figure 1a. First, we prepared MoS_2_ nanolayer on the SiO_2_ surface through ALD. Then, the C_60_ nanolayer was evaporated on MoS_2_ by an evaporation coater. Figure 1f shows the AFM diagram of the 90c-MoS_2_-120s-C_60_ composite nanolayer in the channel. Under the appropriate ALD cycle and evaporation time, a composite nanolayer with good surface morphology could be obtained. Finally, a gold electrode was evaporated on the nanolayer as the source and drain electrode of FET after the photolithography mask, as shown in Figure 1d. It can be seen from Figure 1e that the thickness of the source and drain electrode is 40 nm. Figure 1b shows the Raman characterization spectra of the 90c-MoS_2_, 120s-C_60_, and 90c-MoS_2_-120s-C_60_ composite nanolayers. The MoS_2_ nanolayer prepared on silica substrate has two characteristic peaks near 382 cm^−1^ and 405 cm^−1^, corresponding to two vibration modes of in-plane vibration (E^1^_2g_) and in-plane vibration (A_1g_), respectively [21]. There is a characteristic peak at 273 cm^−1^ in the C_60_ nanolayer obtained by thermal evaporation on the silica substrate, the lowest frequency H_g_ vibration mode. There are two characteristic peaks at 497 cm^−1^ and 1469 cm^−1^, which are two symmetrical vibration modes of A_g_ and A_g_(2), respectively, namely the double bond stretching the vibration and the “breathing” vibration of the five-membered ring in the plane [22]. These characteristic peaks indicate the successful preparation and stable existence of C_60_ nanolayers.

The Raman characteristic peak intensity of the MoS_2_/C_60_ nanolayer is lower than that of the pure MoS_2_ or C_60_ nanolayer. The 1469 cm^−1^ characteristic peak in the MoS_2_/C_60_ sample has a significant red shift compared with the C_60_ sample, indicating that charge transfer has occurred from MoS_2_ to C_60_, forming a potential barrier [23]. According to Figure 1c, 14.2° is the diffraction peak of the MoS_2_ (002) crystal plane, corresponding to the layer spacing of MoS_2_ is 0.62 nm [24]. There are (111), (220), (311), and (222) planes in the C_60_ nanolayer, corresponding to the XRD diffraction peaks of 10.8°, 17.7°, 20.8°, and 21.7°, respectively [25]. The above diffraction peaks can be measured on MoS_2_/C_60_ samples, indicating that the composite nanolayer was successfully prepared and stably existed. The 37° peak of the MoS_2_ sample disappears on the MoS_2_/C_60_ sample, indicating that C_60_ forms a close covering on MoS_2_, and the composite film is well combined.

### 3.2. FET Performance Optimization

The MoS_2_/C_60_ composite nanolayer is deposited on the clean, polished silicon dioxide surface. In ALD deposition, different cycles affect the growth of the MoS_2_ nano layer [20]. When the number of cycles is small, the nucleation sites are dispersed, and MoS_2_ grows island on the silicon dioxide surface, which cannot form a continuous film, resulting in poor surface morphology. When used in FET as channel material, the carrier transfer in the channel is affected due to the intermittence of the nanolayer, which affects the device performance and detection results. When the number of cycles is too large, the nanolayer grains contact each other and induce MoS_2_ to grow longitudinally out-of-plane. With the increase in cycles, the out-of-plane growth finally produces a nanoflower structure [26]. The film thickness cannot be effectively controlled, and the surface morphology is damaged, which affects the preparation and performance of FET devices. Therefore, it is necessary to control the number of ALD cycles to reduce longitudinal growth while ensuring the continuity of MoS_2_ nanolayers.

**Figure 2:**
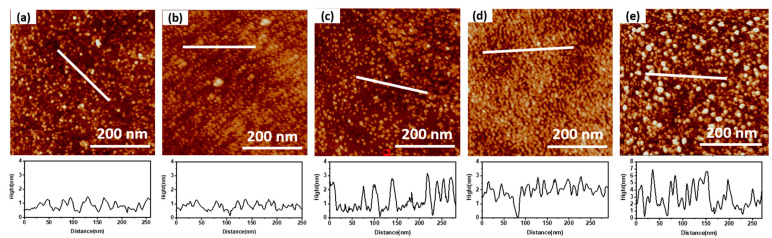
AFM characterization of surface morphology of (**a**) 30c-MoS_2_ nanolayer, (**b**) 45c-MoS_2_ nanolayer, (**c**) 60c-MoS_2_ nanolayer, (**d**) 75c-MoS_2_ nanolayer, and (**e**) 90s-MoS_2_ nanolayer.

Figure 2 shows the surface morphology of MoS_2_ with different ALD cycles. According to the AFM diagram in Figure 2, the film’s roughness at different cycles is calculated to obtain Table 1. MoS_2_ deposited by 30 ALD cycles has independent grains and high surface roughness. Compared with 30 cycles, MoS_2_ with 45 cycles has increased grain size, increased film surface coverage, and reduced surface roughness. At this stage, the MoS_2_ crystal shows island growth. MoS_2_ deposited by 60 cycles ALD continues to grow at the grain boundary to form a new film. However, the continuity of the newly formed film is poor, which increases the surface roughness. The grain size of MoS_2_ at the grain boundary increases, and the surface roughness of the film decreases after 75 cycles. The thickness uniformity of MoS_2_ film prepared by 90-cycle ALD deposition is poor, which may be related to the contact between MoS2 grains and the out-of-plane longitudinal growth, resulting in a sharp increase in the surface roughness of the film.

**Figure 3:**
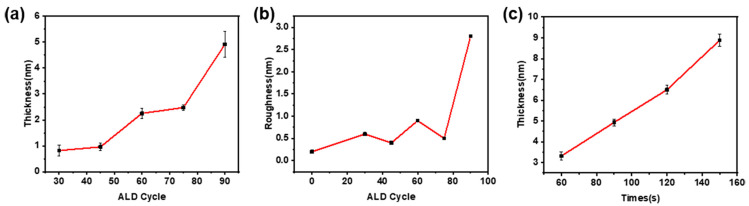
(**a**) The relationship between MoS_2_ thickness and ALD cycle number; (**b**) The relationship between surface roughness of MoS_2_ film and ALD cycle; (**c**) The relationship between C_60_ thickness and hot evaporation time.

Figure 3a shows the relationship between the thickness of MoS_2_ and the number of ALD cycles. Figure 3b shows the broken line diagram between the surface roughness of MoS_2_ film and the number of ALD cycles. It can be seen from Figure 3a that the thickness of MoS_2_ is positively correlated with the number of ALD cycles. The MoS_2_ thickness increases slightly at 30 to 45 cycles and 60 to 75 cycles. The thickness of MoS_2_ from 30 cycles to 45 cycles remains between 0.6 nm and 1.2 nm, which is 1–2 layers of MoS_2_; The thickness of MoS_2_ from 60 cycles to 75 cycles remains between 2 nm and 2.6 nm, which is 3–4 layers of MoS_2_. In these two stages, the thickness of MoS_2_ is basically unchanged, but the film surface coverage and continuity are improved, and the surface roughness is reduced. The change in MoS_2_ film thickness and surface roughness reflects the growth characteristics of MoS_2_ grown by ALD, which are island growth and grain boundary growth.

As shown in Figure 2d, the surface morphology of MoS_2_ deposited by ALD for 75 cycles is uniform and has good continuity, conducive to the fabrication and performance regulation of subsequent FET devices.

**Figure 4:**
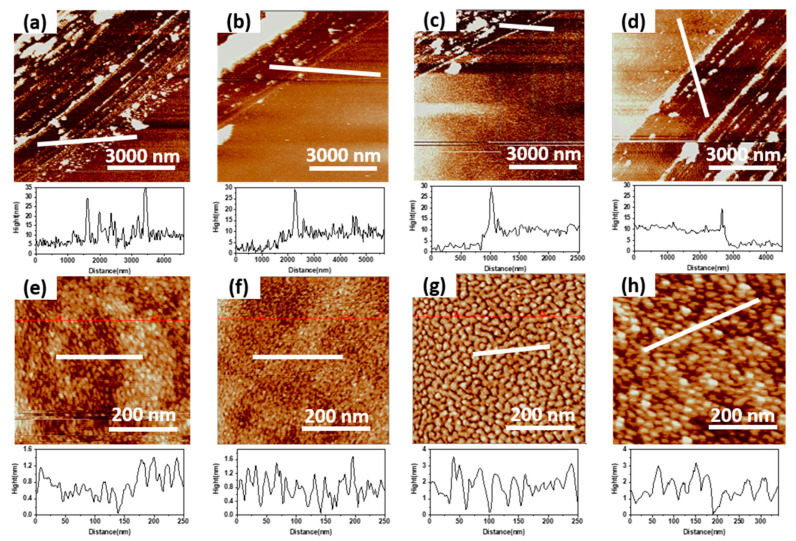
AFM characterization of the thickness of (**a**) 60s-C_60_ nanolayer, (**b**) 90s-C_60_ nanolayer, (**c**) 120s-C_60_ nanolayer, and (**d**) 150s-C_60_ nanolayer after partial removal; AFM characterization of surface morphology of (**e**) 60s-C_60_ nanolayer, (**f**) 90s-C_60_ nanolayer, (**g**) 120s-C_60_ nanolayer, and (**h**) 150s-C_60_ nanolayer.

As shown in Figure 4, the evaporation time affects the thickness and surface morphology of the C_60_ nanolayer. With the increase of evaporation time, the thickness of the nanolayer gradually increases, as shown in Figure 4a–d. As shown in Figure 3c, the thickness of C_60_ is positively correlated with the thermal evaporation time, basically in a linear relationship, and the film-forming rate can be calculated as 0.055 nm/s. As the evaporation is carried out under vacuum conditions, the diameter and surface crystallization of the C_60_ microclusters can be controlled by controlling the atmosphere and vacuum degree of the evaporation environment [27]. As shown in Figure 4e–g, with the evaporation time increasing from 30 s to 120 s, C_60_ microclusters with uniform particle size gradually form on the silica surface, and the microclusters are uniformly distributed to form a film. When the thermal evaporation time is less than or equal to 120 s, the diameter of the C_60_ grain is positively correlated with the evaporation time and finally reaches about 20 nm. Increase the evaporation time again, and the diameter of C_60_ micro clusters does not increase. Still, the irregular stacking of micro clusters is formed on the silicon dioxide surface, and the surface morphology is uneven, as shown in Figure 4h. As shown in Table 2, the average surface roughness of the film increases with the increase of C_60_ thermal evaporation time. When the thermal evaporation time is less than 120 s, the film roughness increases slightly. This is due to the gradual formation of microclusters in C_60_, and the increase in the diameter of the microclusters is the main reason for the increase in the film surface roughness. When the thermal evaporation time reaches 150 s, the uneven stacking of microcluster particles leads to a rapid increase in surface roughness. Therefore, it is necessary to control the evaporation time of C_60_ to 120 s to obtain C_60_ nanolayers with uniform cluster diameter and uniform distribution.

**Figure 5:**
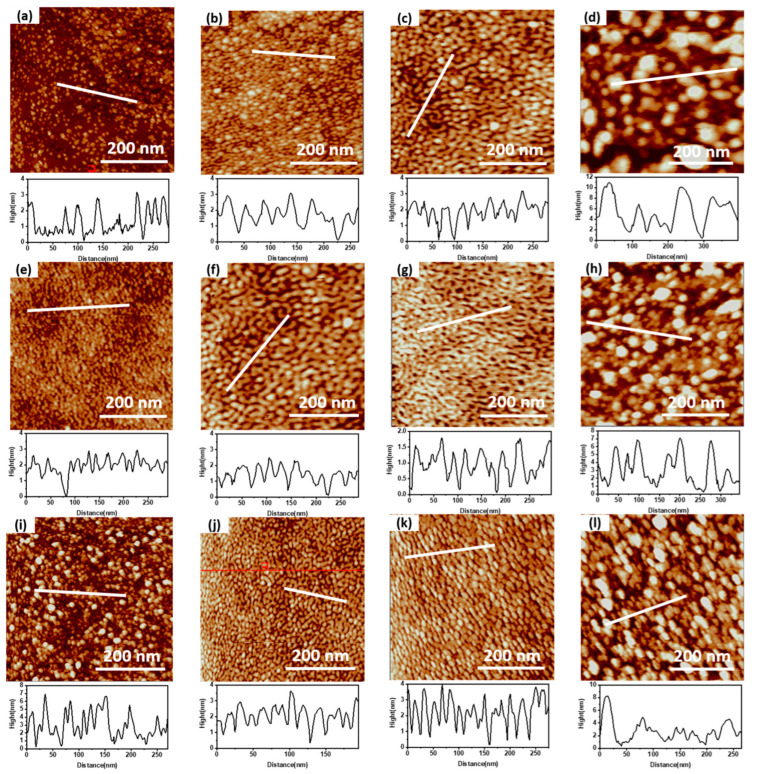
AFM characterization of (**a**) 60c-MoS_2_ nanolayer; (**b**–**d**) 60c-MoS_2_-90s/120s/150s-C_60_ nanolayer; (**e**) 75c-MoS_2_ nanolayer; (**f**–**h**) 75c-MoS_2_-90s/120s/150s-C_60_ nanolayer; (**i**) 90c-MoS_2_ nanolayer; (**j**–**l**) 90c-MoS_2_-90s/120s/150s-C_60_ nanolayer.

The surface morphology of C_60_ evaporated on the MoS_2_ nanolayer is different from that on silicon dioxide, as shown in Figure 5. Under the conditions of maintaining the evaporation environment atmosphere and vacuum degree, the diameters of C_60_ microclusters on the surface of MoS_2_ nanolayers in different cycles are basically consistent, as shown in Figure 5d,h,i. However, due to the uneven stacking of C_60_ evaporated for a long time, the surface roughness of x-MoS_2_-150s-C_60_ is relatively large, as shown in Table 3. The surface morphology of MoS_2_ nanolayers with different cycles is different. The evaporation time required to form C_60_ nanolayers with uniform particle size and a uniform distribution is different, as shown in Figure 5b,c,f,g,j,k. Therefore, as shown in Table 3, the surface roughness of x-MoS_2_-90s/120s-C_60_ is small. The surface morphology of the 75c-MoS_2_-90s/120s-C_60_ and 90c-MoS_2_-90s/120s-C_60_ nanolayers is relatively good.

According to the above conditions, a series of optimized MoS_2_/C_60_ composite nanolayers are selected to fabricate FET devices after mask and evaporation electrode. The device performance is tested through a four-point probe platform. C_60_ has good photoelectric characteristics and large light absorption in the 400 nm to 500 nm range [28]. Therefore, 405 nm blue-violet light is selected to irradiate the channel area of FET to improve the device’s performance.

**Figure 6:**
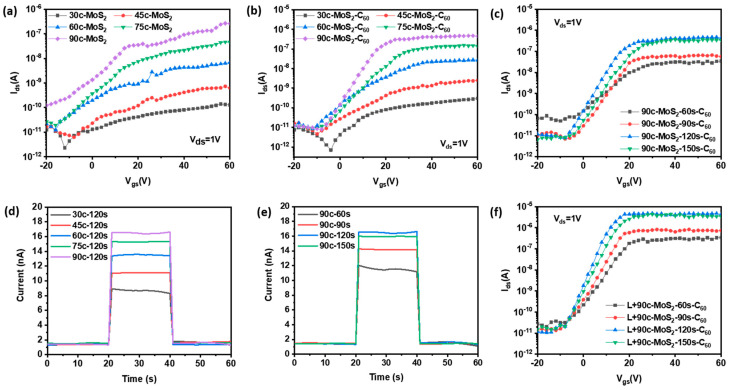
(**a**) FET transfer characteristic curve with different MoS_2_ cycles; (**b**) 120s-C_60_-FET transfer characteristic curve with different MoS_2_ cycles; (**c**) 90c-MoS_2_-FET transfer characteristic curve with different C_60_ evaporation duration; (**d**) Photoelectric characteristics of 120s-C_60_ channel materials with different MoS_2_ cycles, (**e**) Photoelectric characteristics of 90c-MoS_2_ channel materials with different C_60_ evaporation duration, and (**f**) different C_60_ duration 90c-MoS_2_ FET transfer characteristic curves under 405 nm blue-violet light.

The transfer characteristic curves of different MoS_2_/C_60_-FETs are shown in Figure 6a–c. Figure 6a shows the transfer characteristic curve of MoS_2_-FET with a different number of ALD cycles. With the increase of the ALD cycles, the number of MoS_2_ layers and the FET on-off ratio increase. Figure 6b,c shows the transfer characteristic curves of x-cycles-MoS_2_-120s-C_60_-FET and 90c-MoS_2_-x-second-C_60_-FET, respectively. The on-off ratio data of 90c-MoS_2_-120s-C_60_-FET is the best, reaching 5.8 × 10^4^. Due to the photoelectric characteristics of C_60_, 405 nm blue-violet light is irradiated on the channel material. According to Figure 6d,e, the optical current of 90c-MoS_2_-120s-C_60_ channel material increases the most. According to Figure 5, the increase of MoS_2_ cycles increases the number of nanolayers, which is conducive to the current conduction of FET. The film of C_60_ is the most uniform when it is evaporated for 120 s. AFM diagram shows that the 90c-MoS_2_-120s-C_60_ composite has the best surface morphology, and the test results show it has the best FET device performance. The maximum photocurrent gain is obtained when the blue-violet light is increased. It can be seen from Figure 6f that the switch ratio of 90c-MoS_2_-120s-C_60_-FET increases by nearly an order of magnitude under the light condition, reaching the maximum value of 4.36 × 10^5^. 

### 3.3. Ultrasensitive DETECTION of miRNA-155

**Figure 7:**
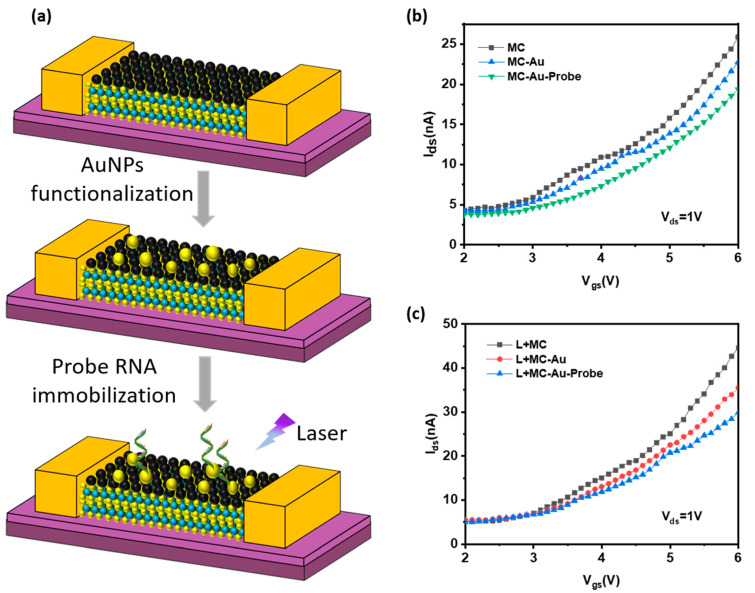
(**a**) Preparation steps of FET biological detection platform: hot evaporate AuNPs on the channel, fix Probe RNA on the AuNPs, and irradiate the channel with 405 nm blue-violet light to improve the FET performance; (**b**) Without an external light source, the I_d_–V_g_ curve of MoS_2_/C_60_ (MC)-FET, the I_d_–V_g_ curve after hot evaporation of AuNPs, and the I_d_–V_g_ curve after fixed Probe RNA; (**c**) I_d_–V_g_ curve of each step under 405 nm blue-violet light.

The construction process of the MoS_2_/C_60_-FET biological detection platform is shown in Figure 7a. First, AuNPs were deposited on the MoS_2_/C_60_ (MC) channel surface through evaporation. The current response of the device’s transfer curve (I_d_–V_g_) decreased after deposition, as shown in Figure 7b,c. Then, probe RNA is fixed on AuNPs through an Au-S bond to capture the target miRNA. After fixing Probe RNA, the transfer curve current response of the device also decreased. This is because the deposition of AuNPs and the immobilization of Probe RNA led to the p-type doping of channel materials [11]. At the same time, it can be seen from Figure 7b,c that adding 405 nm blue-violet light to the channel material could effectively improve the current response of the device’s transfer curve.

**Figure 8:**
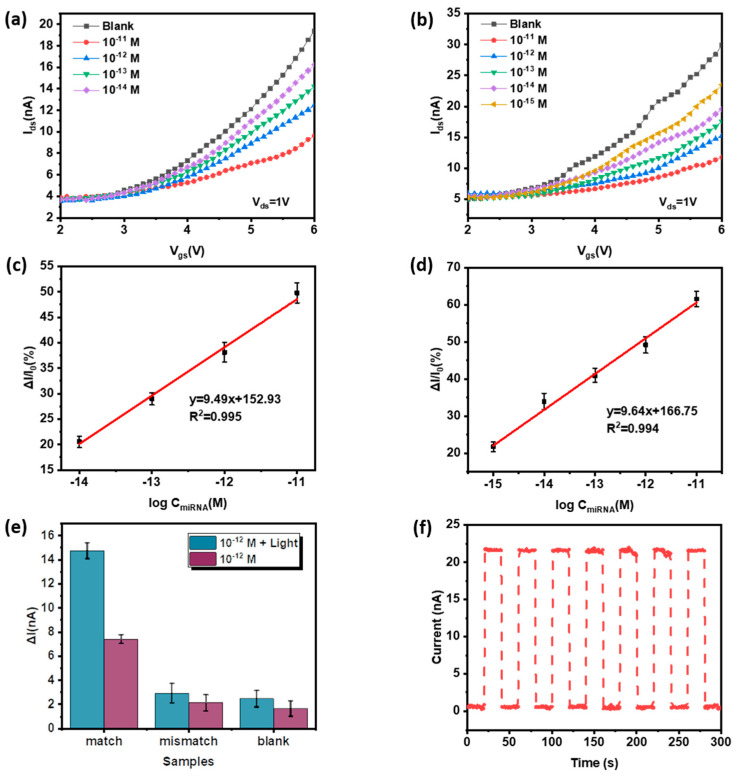
(**a**) Transfer curve current response of Blank (MC-Au-Probe) and target miRAN-155 added with 10^−11^ M, 10^−12^ M, 10^−13^ M, 10^−14^ M in PBS (pH 7.4, 0.01 M) solution without external light, and corresponding calibration curve (**c**); (**b**) Under the condition of 405 nm blue-violet light, in PBS (pH 7.4, 0.01 M) solution, Blank (MC-Au-Probe) and 10^−11^ M, 10^−12^ M, 10^−13^ M, 10^−14^ M, 10^−15^ M target miRAN-155 transfer curve current response, and the corresponding calibration curve (**d**); (**e**) 10^−12^ M target miRNA-155, single base mismatch miRNA-155 and blank control group’s current difference between the transfer curve and Blank (MC Au Probe) under the conditions of external light and no external light; (**f**) Repeatability experiment of the device in 300 s.

Figure 8 shows the detection results of target miRNA-155 by the FET biological detection platform. Among them, the current response of the transfer curve of the Blank (MC-Au-Probe) device is the largest, and the target miRNA is captured with Probe RNA. The higher the concentration of target miRNA is, the greater the current in the device transfer curve decreases. The lower the concentration is, the closer the device transfer curve current is to Blank’s current. The detection range of FET without external light is 10 fM–10 pM; The detection range of FET under 405 nm blue-violet light is 1 fM–10 pM. Since the charge is transferred from C_60_ to MoS_2_ at the contact interface of the composite, and C_60_ has good blue light absorption and photoelectric performance, the addition of a laser improves the detection range of the device [29]. The linear fit R^2^ of the collaborative calibration is 0.995 and 0.994 without the external light and the external blue-violet light, respectively, indicating that the two kinds of detection have high accuracy. According to the formula:

(1) $$y=9.49\;\log C_{miRNA}+152.93$$

(2) $$y=9.64\;\log C_{miRNA}+166.75$$

y = (I_ds_ − I_Blank_)/I_Blank_, y based on blank sample data to avoid external interference; C: concentration of target miRNA-155, the detection limit without an external light source is 78.81 am and the detection limit under the condition of applied light is 5.16 aM. The detection limit is increased by adding blue-violet light. Through the detection of single base mismatched miRNA-155, compared with the target miRNA-155, as shown in Figure 8e, the current difference of single base mismatched transfer curve is significantly lower than the target miRNA, proving that the biological detection platform of this work has good selectivity specificity. In addition, repeated detection experiments were carried out on the biological detection platform of this work for 300 s, as shown in Figure 8f. The current stability of repeated experiments was good, which proves that the biological monitoring platform of this work has good repeatability based on blank sample data to avoid external interference. To sum up, this detection platform has good selective specificity and repeatability for miRNA-155 detection under conditions of blue and violet light. The detection range is 1 fM–10 pM, and the detection limit is 5.16 aM. Compared with previous work, MoS_2_/C_60_-FET has a lower detection limit for detecting miRNA-155 under applied light (Table 4).

## 4. Conclusions

The C_60_/MoS_2_ composite commonly used in solar cells was used in the FET channel. Detecting miRNA-155 was achieved by evaporating and depositing AuNPs and connecting Probe RNA with the Au-S bond. The thickness of the MoS_2_/C_60_ composite nanolayer was controlled by the ALD cycle and the thermal evaporation time. According to AFM characterization, the composite nanolayer 90c-MoS_2_-120s-C_60_ with good surface morphology was used as channel material to make FET. Due to the electron transfer between MoS_2_/C_60_ composite nanolayers, combined with the excellent photoelectric properties of C_60_, the performance of FET was significantly improved by irradiating the channel material with a 405 nm laser. The linear detection window of the obtained biological detection platform for miRNA-155 was 1 fM–10 pM, and the minimum detection limit was 5.16 aM. 

This research has developed the application of C_60_ in the field of FET, providing more possibilities for the future development of FET and ultra-sensitive biological detection. Based on this paper, we can explore more applications of C_60_-related heterojunctions in the field of FET.

## Figures and Tables

**Table 1:** Average surface roughness of MoS_2_ under different ALD cycles.

	30c-MoS_2_	45c-MoS_2_	60c-MoS_2_	75c-MoS_2_	90c-MoS_2_
Ra	0.2	0.3	0.5	0.7	1.5

**Table 2:** Average surface roughness of C_60_ with different thermal evaporation times.

	0s-C_60_	60s-C_60_	90s-C_60_	120s-C_60_	150s-C_60_
Ra	0.2	0.3	0.5	0.7	1.5

**Table 3:** Average surface roughness of MoS_2_/C_60_ composite film.

Ra	90s-C_60_	120s-C_60_	150s-C_60_
60c-MoS_2_	0.6	0.6	3.4
75c-MoS_2_	0.4	0.5	3.2
90c-MoS_2_	0.4	0.7	2.0

**Table 4:** Comparison of the analytical performance of microRNA-155 detection platforms.

Method	Detection Limit	Linear Range
Amperometry	1.87 pM	5.6 pM–0.56 uM [30]
OSWV	12 fM	50 fM–30 pM [31]
Differential pulse voltammetry	0.6 fM	2 fM–8 pM [32]
EIS	5.7 aM	10 aM–1.0 nM [33]
MoS_2_-FET	0.03 fM	0.1 fM–10 nM [19]
MoS_2_/C_60_-FET	5.16 aM	1 fM–10 pM (This work)

## Data Availability

Not applicable.

## Appendix A

Fullerene (C_60_) was purchased from Aladdin Chemical Reagent Co., Ltd. (Shanghai, China). Molybdenum pentachloride (MoCl_5_) and phosphate buffer solution (PBS) were purchased from Sigma Aldrich (Shanghai) Trading Co., Ltd. (Shanghai, China). Positive photoresist (RZJ-304), positive photoresist developer (RZX-3038), and positive photoresist stripper (RBL-3368) were purchased from Suzhou Ruihong Electronic Chemicals Co., Ltd. (Suzhou, China). Pure gold particles (Au99.999) were purchased from Fuzhou INFI Fast Optoelectronic Technology Co., Ltd. (Fuzhou, China). The target miRNA-155, probe miRNA, and related nucleic acid sequences were processed by Shanghai Sangong Biotechnology Co., Ltd. (Shanghai, China). The specific base sequence is:

Probe miRNA-155: 5′-NH_2_-AAA AAA AAC CCC UAU CAC GAU UAG CAU UAA-3′.

Target miRNA-155: 5′-UUA AUG CUA AUC GUG AUA GGG GU-3′.

One-base mismatch miRNA-155: 5′-UUA AUG CUA CUC GUG AUA GGG GU-3′.
